# Noninvasive longitudinal assessment of early-stage Duchenne muscular dystrophy: *In vivo* diaphragm imaging in *mdx* mice

**DOI:** 10.1016/j.ultras.2025.107846

**Published:** 2025-10-09

**Authors:** Jeehyun Lee, Nia O. Myrie, Woojin M. Han, Young C. Jang, Andrés J. García, Stanislav Emelianov

**Affiliations:** a School of Electrical and Computer Engineering, Georgia Institute of Technology, Atlanta, GA, USA; b Wallace H. Coulter Department of Biomedical Engineering, Georgia Institute of Technology and Emory University School of Medicine, Atlanta, GA, USA; c Department of Orthopedics, Icahn School of Medicine at Mount Sinai, New York, NY, USA; d Department of Orthopedics, Emory Musculoskeletal Institute, Emory School of Medicine, Atlanta, GA, USA; e George W. Woodruff School of Mechanical Engineering, Georgia Institute of Technology, Atlanta, GA, USA

**Keywords:** Elastography, Shear wave elasticity imaging, Phase velocity, Viscoelastic, Muscular dystrophy, Diaphragm, DMD

## Abstract

Duchenne muscular dystrophy (DMD) is characterized by progressive muscle degeneration, with respiratory muscle weakness significantly impacting patient outcomes. Early detection of disease progression is critical for evaluating treatment strategies. This study investigates the feasibility of ultrasound shear wave elasticity imaging (US-SWEI) for assessing early-stage respiratory muscle changes in *mdx* mice, a model for DMD, aged 3 to 6 months. Longitudinal *in vivo* imaging evaluated the diaphragm’s viscoelastic properties, with group and phase velocities measured to capture biomechanical changes. In *mdx* mice, group velocity increased from 3.47 ± 0.15 to 4.20 ± 0.20 m/s, whereas wild-type values changed only modestly (2.76 ± 0.11 to 3.01 ± 0.16 m/s). Histological analysis confirmed a significant positive correlation between group velocity and collagen deposition (*R*^2^ = 0.54*, P* = 0.025), supporting fibrosis as a primary factor driving changes in diaphragm viscoelastic properties. These findings establish US-SWEI as a noninvasive, feasible approach for monitoring respiratory muscle health and advancing preclinical DMD research. By identifying early-stage changes in diaphragm properties, this approach enables the development of therapeutic interventions targeting respiratory complications. Furthermore, US-SWEI presents a potential avenue for assessing and monitoring neuromuscular diseases.

## 1. Introduction

Duchenne muscular dystrophy (DMD) is a genetic muscle-wasting disorder caused by mutations in the dystrophin gene, resulting in muscle degeneration and fibrosis. It is the most common inherited muscular dystrophy, affecting approximately 1 in 5000 males worldwide and leading to progressive loss of mobility and respiratory function [1, 2]. Because strengthening these muscles could significantly improve breathing capacity and even extend life, researchers are increasingly focusing on respiratory function as a key area of treatment. Current therapies aim not only to slow the disease but also to support muscle strength, particularly within the respiratory system, to improve overall quality of life.

Various therapeutic approaches are being explored, including gene editing to correct genetic defects [3–5] and the use of viral vectors to deliver functional dystrophin genes to muscle cells [6]. However, these therapies face significant hurdles, such as immune responses that can cause toxicities and complications [7,8]. Many patients have pre-existing antibodies that neutralize the vectors, limiting their eligibility for treatment. Moreover, gene therapies are less effective in older patients who have advanced disease stages with significant muscle wasting and cardiac complications [9,10]. Additionally, gene therapy’s potential as a one-time treatment is often compromised due to immune clearance, necessitating repeated administrations to maintain effective gene expression [10]. Given these limitations, early detection and monitoring of disease progression are crucial for evaluating treatment efficacy and guiding therapeutic strategies.

Ultrasound shear wave elasticity imaging (US-SWEI) [11] has been widely used to assess tissue mechanical properties, including diaphragm function, by providing quantitative measurements of shear modulus [12–18]. While SWEI has been extensively applied in clinical diaphragm assessment, its use in DMD research has largely focused on limb muscles, with limited studies on diaphragm mechanics [19–23]. Our previous research demonstrated the effectiveness of US-SWEI in tracking disease progression in *mdx* mice aged 6–12 months, showing strong correlations between wave speed, muscle fibrosis, and fiber size [24]. Building on this foundation, this study evaluates the feasibility of US-SWEI for detecting early-stage disease progression in *mdx* mice aged 3–6 months. We hypothesize that US-SWEI can serve as an applicable, noninvasive tool for assessing diaphragm viscoelastic properties, offering a quantitative approach to monitoring respiratory muscle health. Understanding early-stage biomechanical changes in the diaphragm is essential for developing effective interventions and improving preclinical evaluation of DMD treatments.

## 2. Methods

### 2.1. Animal subjects and study design

All experimental procedures performed on mice were approved by the Institutional Animal Care and Use Committee of the Georgia Institute of Technology. Five *C57Bl/6J* (WT) male mice were used as controls, and four dystrophin-deficient *B6Ros.Cg-Dmd*^*mdx−4Cv*^/J (*mdx*) male mice were monitored longitudinally from 3 to 6 months of age. To increase the sample size at the earliest time point, three additional *mdx* mice (3 months old) were added specifically to AgeGroup G1. These additional animals were included to ensure sufficient baseline data, as slight differences in date of birth prevented their longitudinal follow-up across later time points. Each group underwent imaging at four time points, denoted as AgeGroup G1 through G4, representing the age ranges of 3–3.2 months old (G1), 3.5–3.8 months old (G2), 4–4.2 months old (G3), and 5–6 months old (G4).

### 2.2. In vivo ultrasound imaging setup

The imaging setup followed the protocol reported by [24], utilizing a Vantage 128 ultrasound imaging system with a CL15–7 linear array transducer (Verasonics, Inc., Kirkland, WA, USA). Shear waves (SWs) were induced and tracked using acoustic radiation force impulses (200 *μ*s at 9 MHz) and plane wave imaging (9 MHz center frequency, 8 kHz frame rate) [25]. For each acquisition, successive unfocused plane waves were transmitted and coherently compounded, producing high-frame rate image sequences suitable for capturing transient shear wave motion. Mice were anesthetized with 2% isoflurane in 0.6 L/min oxygen.

### 2.3. Ultrasound shear wave elasticity imaging

**Fig. 1.**
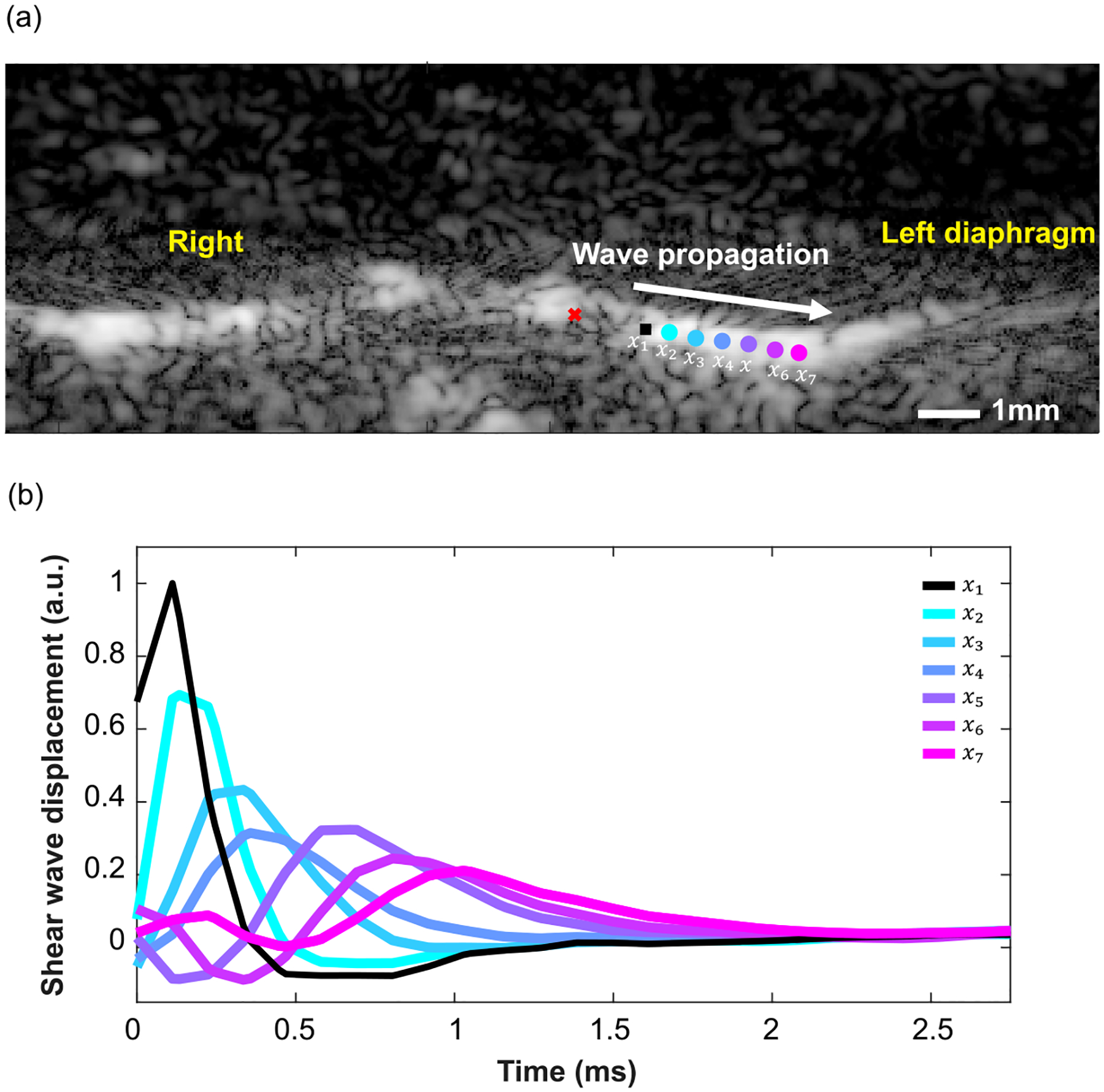
(a) *In vivo* B-mode image of the mouse diaphragm. (b) Shear wave propagation in the left diaphragm segment. Group velocity is estimated between *w*_1_ and *w*_2_ from their arrival times, and phase velocity is determined from the phase shift.

US-SWEI data were acquired from three distinct imaging planes in each mouse, with shear waves generated on the left side of the diaphragm (Fig. 1). To ensure consistency across measurements, all pushing and imaging acquisitions were performed within a short late-exhalation window, corresponding to the most relaxed phase of the respiratory cycle. This approach minimized respiratory motion artifacts and stabilized diaphragm positioning. After the push beam excited the tissue and shear waves (SWs) began propagating, SW motion data from two points, separated by a lateral distance *d* (2.3 to 3.0 mm), were used to estimate velocity. The axial displacement of tissue was extracted from beamformed in-phase and quadrature (IQ) data using Loupas’ 2D autocorrelator [26], which computes phase shifts between consecutive imaging frames within a defined axial window. Displacement estimation was performed over an axial window of 0.52 mm, which closely corresponds to the reported diaphragm thickness in mice across various models [27,28], ensuring that small regional variations in thickness do not significantly affect velocity estimates. The resulting displacement fields were then used to analyze shear wave motion.

Given the diaphragm’s structure and dispersive properties, shear waves exhibit both group velocity and frequency-dependent phase velocity variations. To account for this, both were estimated from the displacement data. Group velocity (*v*_*g*_) was estimated using a cross-correlation method [29], which aligns the received signal with a reference signal to determine the time lag corresponding to peak correlation, thereby marking the wave’s arrival. Original data were sampled at 8.6 kHz (*Δt* = 116 *μ*s) and interpolated to a temporal resolution of 37.38 *μ*s per sample. The analysis used the full 78-sample interpolated signals, corresponding to a kernel extent of 2.91 ms. Each *v*_*g*_ value within the segment was measured individually, and the median value of these measurements was calculated to represent the segment’s group velocity.

To extract phase velocity (*v*_*p*_), we employed the wavelet synchrosqueezing transform (SST). Conventional approaches often rely on the two-dimensional Fourier transform (2D-FT) to analyze spatiotemporal shear wave propagation in k-space [30]. While effective, 2D-FT and similar methods [31–34] require dense lateral sampling, making them less suitable when propagation distances are short. Wavelet-based techniques, particularly the continuous wavelet transform (CWT), have been applied to phase velocity estimation in various scientific domains [35,36] and subsequently adapted for shear wave analysis in biological tissues [37]. While CWT compensates for limited spatial sampling, its resolution is constrained by wavelet shape, leading to spectral smearing and leakage [38–40]. SST refines the CWT by redistributing spectral energy to true frequency locations, thereby improving time–frequency resolution and enabling precise tracking of frequency-dependent velocity variations [41–43]. This makes SST particularly well-suited for analyzing non-stationary signals, such as shear waves in skeletal muscle. Phase velocity curves were derived by analyzing phase shifts across the time–frequency representations of signals recorded at two lateral positions. The velocity at each frequency *f* was calculated as

$$v_{p}(f)=\frac{d\cdot \Delta \phi (f)}{2\pi f}$$

where *d* is the lateral separation between the two measurement points, and *Δϕ*(*f*) is the phase shift of the shear wave at frequency *f*. This implementation utilized the Wavelet Toolbox available in MATLAB 2024a (The MathWorks, Inc., Natick, MA, USA) to compute the synchrosqueezed time–frequency representation. Further details on the phase velocity estimation method, including its implementation and validation, are provided in the Supplementary Material.

### 2.4 Histology

After euthanasia, left hemi-diaphragms were explanted, fixed in 4% paraformaldehyde, and then placed in 20% sucrose solution (in PBS) overnight at 4°C. Diaphragm strips were cut with a scalpel and mounted on cork with needles and Tissue-Tek O.C.T. (Sakura Finetek, Torrance, CA, USA), then flash frozen in isopentane cooled with liquid nitrogen. Cross-sections (10 *μ*m) were cut from frozen diaphragms using a cryostat microtome (CryoStar NX70; Thermo Fisher Scientific, Waltham, MA, USA). Frozen muscle sections were stored at −80°C until staining. Muscle cryosections were stained with Gomori’s Trichrome (Polysciences, Inc.; Warrington, PA, USA) for collagen deposition. Representative brightfield images were taken using a 20X/0.8 NA objective lens on an inverted microscope (AxioObserver Z1; Carl Zeiss Microscopy GmbH, Jena, Germany). Brightfield images of whole sections were also taken using a digital microscope (ImageXpress Pico; Molecular Devices, LLC, San Jose, CA, USA). Percentage of collagen deposition was quantified using ImageXpress Pico images by analyzing Gomori’s Trichrome-stained images in FIJI-ImageJ (National Institutes of Health, Bethesda, MD, USA). The “Color Deconvolution” plugin was used to identify collagen-rich areas in the tissue. Deconvolved images were subjected to binarized thresholding and converted to masks. Particle analysis was then used to measure the area of collagen and total tissue area, and the collagen area was divided by total tissue area to calculate the percentage of collagen as a measure of fibrosis.

### 2.5. Statistical analysis

Linear mixed-effects (LME) models were used due to their suitability for analyzing longitudinal data with repeated measurements. With multiple measurements taken from the same mice at different time points, LME models incorporated both fixed effects (AgeGroup) and random effects (MouseID) to account for individual variability. The dependent variables, shear wave velocities (*v*_*g*_ and *v*_*p*_), were modeled as functions of AgeGroup with a random intercept for each MouseID. An unstructured covariance structure was assumed for the random effects. Fixed effect results are reported as estimates, accompanied by standard errors (SE), *P*-values, and 95% confidence intervals (CIs). Pairwise group comparisons were conducted using contrast tests applied to the fixed-effect structure of the LME model. These contrasts tested differences between estimated marginal means of each group, providing sensitivity to group-level differences that may not be fully reflected in the fixed-effects summary. A significance level of *P* <0.05 was considered statistically significant. Correlation analysis was performed to assess relationships between shear wave velocities and collagen percentage, as well as between *v*_*g*_ and *v*_*p*_. Linear regression analysis was also conducted to evaluate the relationship between *v*_*g*_ and *v*_*p*_. The Anderson–Darling test was applied to the residuals of each LME model to verify the assumption of normality. For histological data, Welch’s ANOVA with Dunnett’s T3 multiple comparisons test was used to compare across groups, implemented using GraphPad Prism (version 9.4.1.681).

## 3. Results

### 3.1 Group velocity results

**Fig. 2.**
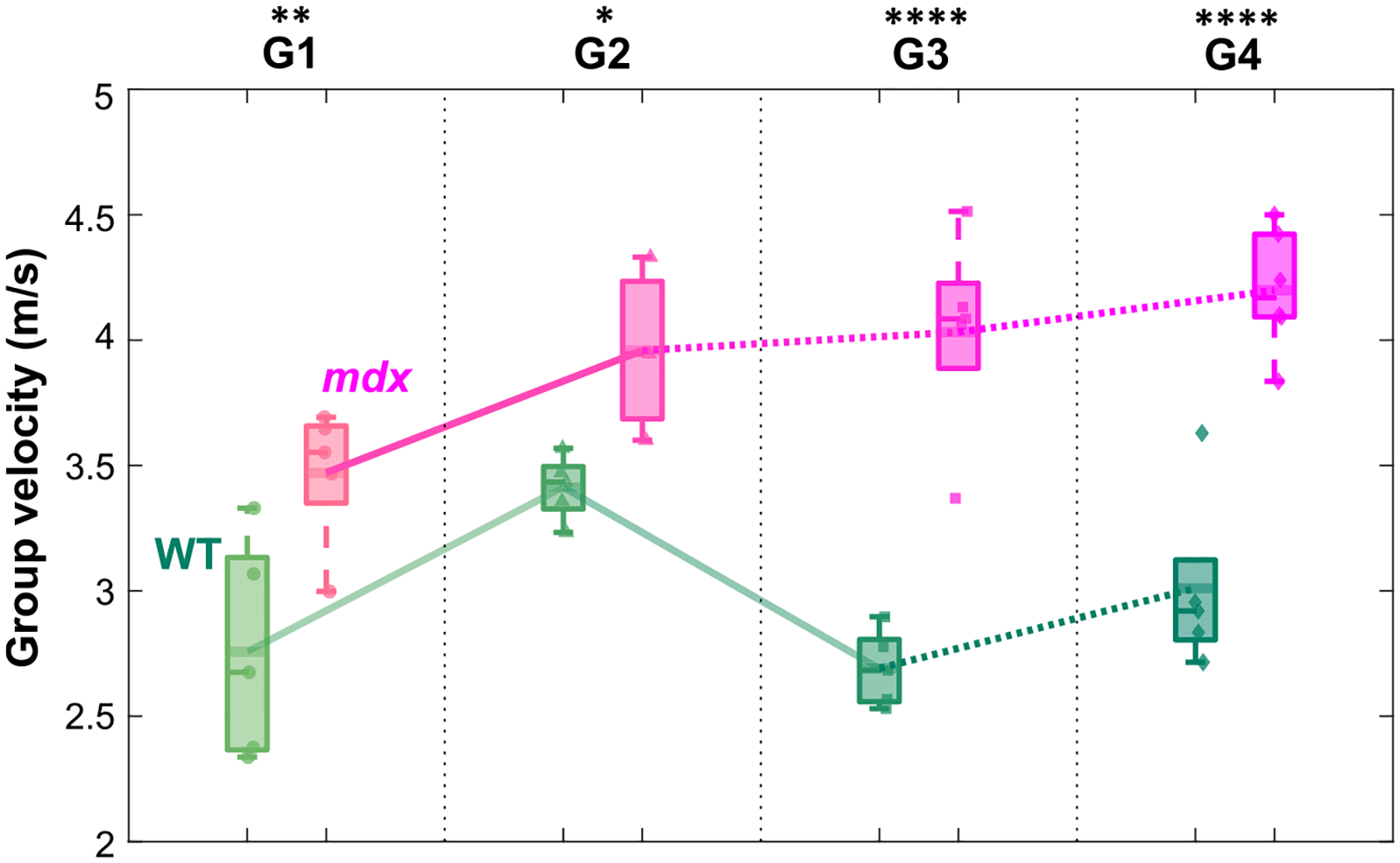
Group velocity (m/s) comparison between WT and *mdx* mice across different age groups (G1 to G4). Box plots display group velocity distributions with individual data points overlaid. Age groups are labeled G1, G2, G3, and G4. WT (green) and *mdx* (pink) mice are compared. Significant age effects are observed in G2, G3, and G4 compared to G1 for both WT and *mdx* mice. Group velocity remains consistently higher in *mdx* mice across all age groups, with each difference statistically significant. Solid lines between adjacent boxes indicate statistical significance, while dotted lines indicate non-significance. * *P* < 0.05, ** *P* < 0.01, *** *P* < 0.001, **** *P* < 0.0001.

The LME model analysis of WT group velocities revealed significant variations across AgeGroup (Table 1, Fig. 2; green). The baseline group (G1) had an estimate of 2.76 m∕s (*P* < 0.0001), serving as the reference point for subsequent comparisons. The estimated group velocity increased to 3.41 m∕s in G2 (*P* = 0.0002), decreased to 2.69 m∕s in G3 (*P* = 0.684), and rose again to 3.01 m∕s in G4 (*P* = 0.124). While the LME model did not identify significant differences for G3 or G4, pairwise comparisons indicated that both G1 vs. G3 and G1 vs. G4 were statistically significant (*P* < 0.0001). In contrast, the increase from G3 to G4 (*P* = 0.05) did not meet the threshold for statistical significance. These results suggest that the changes in group velocity across age groups likely reflect normal growth-related alterations in muscle properties. The increase from G1 to G2, followed by a decrease in G3, points to the possibility of nonlinear growth dynamics, in which developmental changes do not progress uniformly over time.

Following the analysis of WT group velocities, the data for the *mdx* group also exhibited significant differences across AgeGroup (Table 2, Fig. 2; pink). Starting with the baseline group (G1), which had an estimated group velocity of 3.47 m∕s (*P* < 0.0001), subsequent groups showed increases to 3.96 m∕s in G2 (*P* = 0.051), 4.03 m∕s in G3 (*P* = 0.011), and 4.20 m∕s in G4 (*P* = 0.0007). While the LME model did not detect a statistically significant difference between G2 and G1, the result was marginal (*P* = 0.051), suggesting a potential trend. In contrast, pairwise comparisons identified this increase as statistically significant (*P* < 0.0001). Both G3 and G4 showed significant increases relative to G1 in the LME model (*P* = 0.011 and *P* = 0.0007, respectively), consistent with the pairwise comparisons (*P* < 0.0001 for both). Comparisons between G2 and G3 (*P* = 0.766) and G3 and G4 (*P* = 0.416) did not show significant differences. WT and *mdx* groups both exhibited age-related changes in group velocity, with early-stage differences (G1–G2) being the most prominent. While WT mice showed a nonlinear trajectory characterized by a transient decrease in G3, *mdx* mice displayed a more monotonic increase across age groups. In light of the developmental pattern observed in WT mice, the increases in *mdx* mice are likely influenced by a combination of natural growth and disease progression.

Table 3 compares WT and *mdx* velocities by AgeGroup, showing that WT values were consistently lower than *mdx* values across all ages. The between-group differences became more pronounced at G3 and G4, suggesting that disease progression accentuates mechanical divergence with age. Building on the group-specific analyses, we performed a combined LME analysis including AgeGroup, MouseType, and their interaction as fixed effects, with random intercepts for MouseID. The model revealed significant main effects of AgeGroup (*P* = 0.0008) and MouseType (*P* = 0.0002), as well as a significant AgeGroup × Mouse-Type interaction (*P* = 0.014), indicating that age-related trajectories differed between WT and *mdx* groups. Estimated marginal means and full model outputs are provided in Table S1–3 in the Supplementary Material. These findings align with the group-specific analyses and support the interpretation that the progressive increase in *mdx* mice reflects both age-related growth and disease-associated remodeling. Residuals from all group velocity models passed the Anderson–Darling normality test (*P* > 0.05), indicating that the normality assumption of the LME models was met.

### 3.2 Phase velocity results

**Fig. 3.**
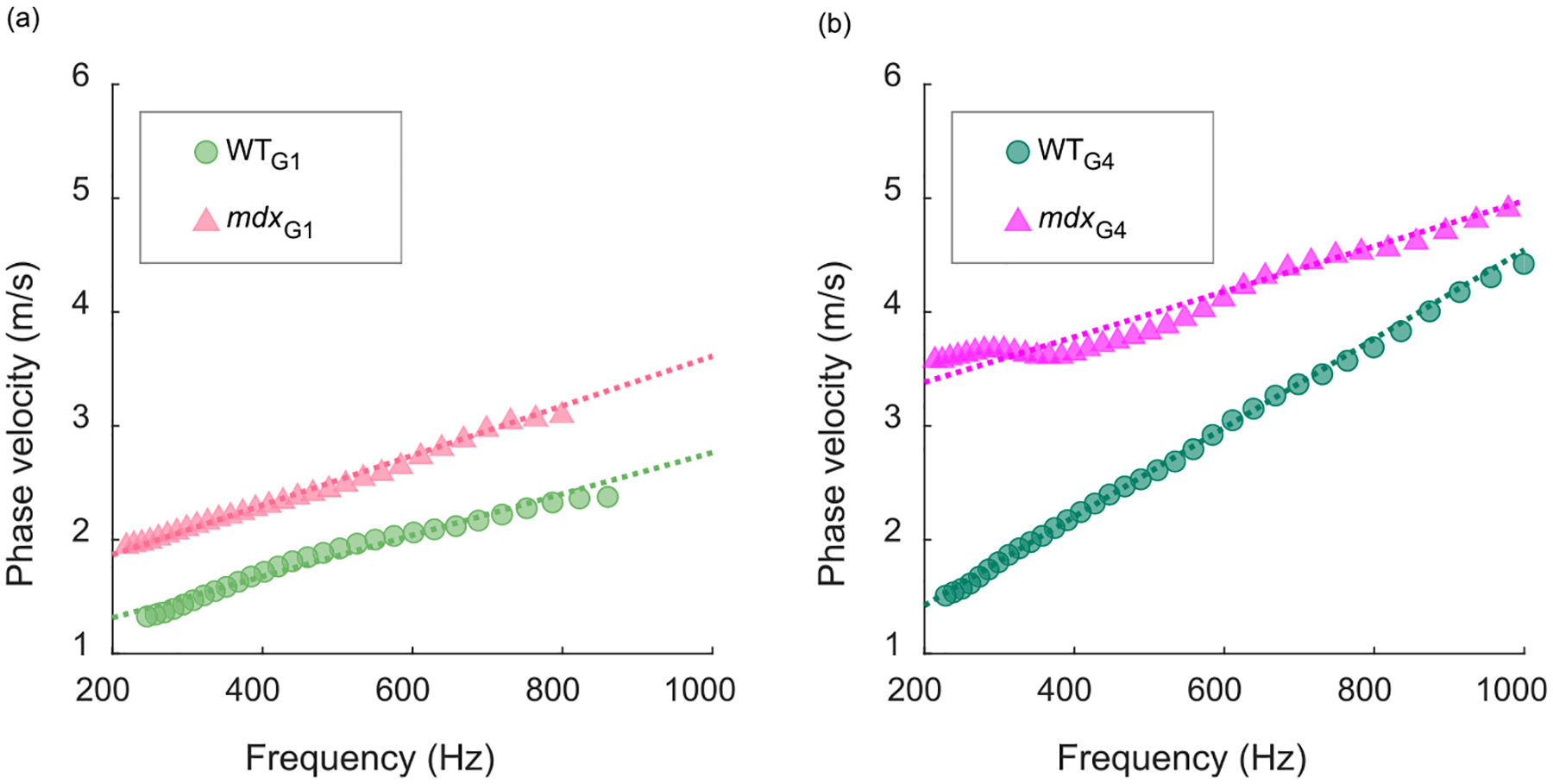
Representative phase velocity measurements for WT and *mdx* mice in age group (a) G1 and (b) G4. Data points (WT: green circles, *mdx*: pink triangles) represent phase velocities retrieved in a defined set of frequency bands defined by 6-dB attenuated response of each shear wave signal. Dashed lines indicate the linear fit used to estimate the dispersion slope.

**Fig. 4.**
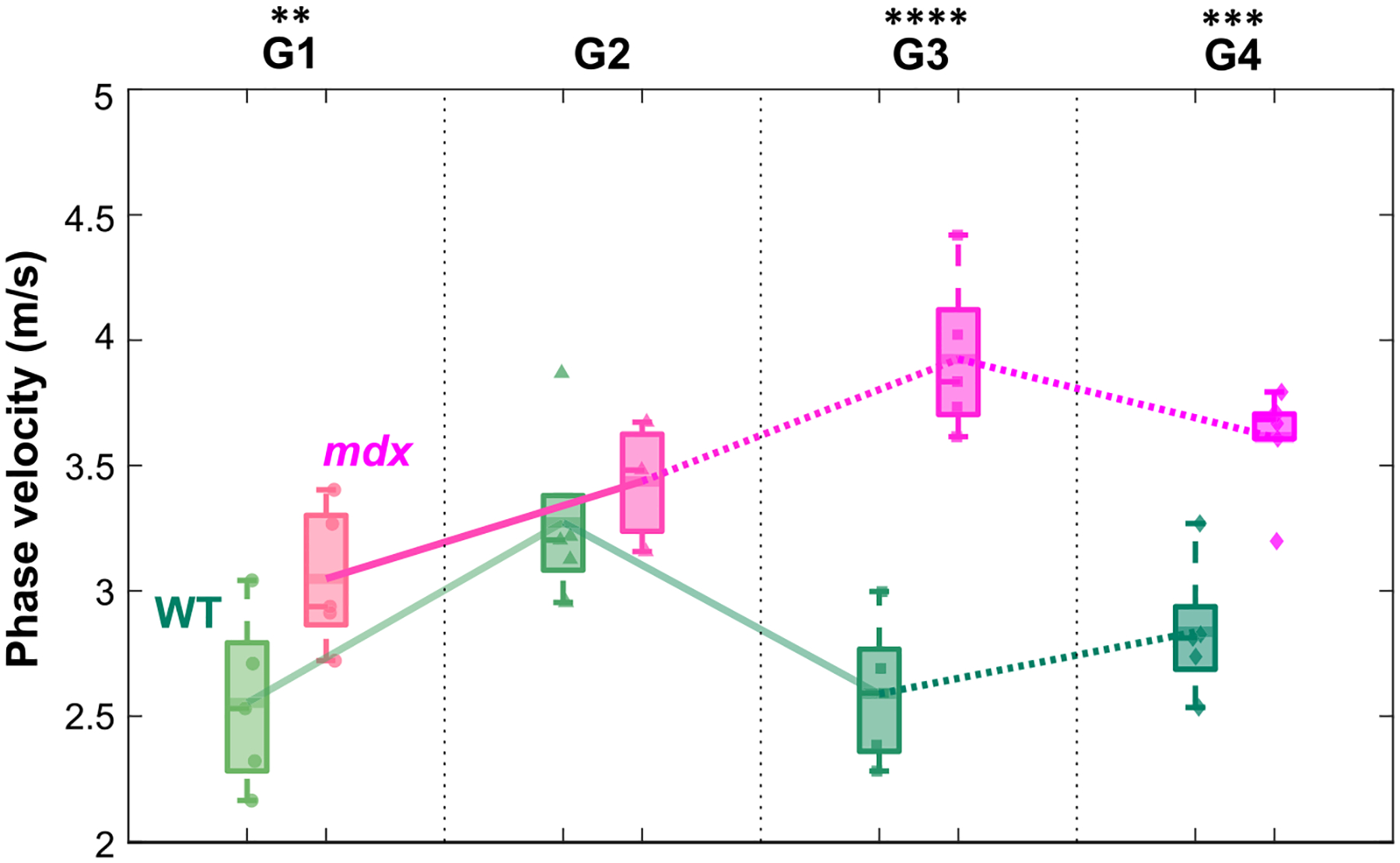
Phase velocity (m/s) comparison between WT and *mdx* mice across different age group (G1 to G4). Each scatter point represents the mean phase velocity in 600–900 Hz frequency band, calculated from individual phase velocity curves. Box plots display group velocity distributions with individual data points overlaid. Age groups are labeled G1, G2, G3, and G4. WT (green) and *mdx* (pink) mice are compared. Phase velocity remains consistently higher in *mdx* mice across all age groups, with each difference statistically significant. Solid lines between adjacent boxes indicate statistical significance, while dotted lines indicate non-significance. * *P* < 0.05, ** *P* < 0.01, *** *P* < 0.001, **** *P* < 0.0001.

Representative phase velocity curves, extracted within frequency bands defined by the 6-dB attenuation threshold of each shear wave signal, are shown in Fig. 3. For consistent comparison across samples, a standardized frequency band (600–900 Hz) was selected based on the average center frequency and upper bound values across all shear wave data. Phase velocity data were linearly fit to mitigate the influence of potential outliers, and the mean phase velocity for each condition was subsequently calculated within this frequency band (Fig. 4). Phase velocity trends were largely consistent with those observed for group velocities, showing significant age-related differences in both WT and *mdx* mice, particularly between G1 and later age groups. Detailed results are presented in Tables 4–6.

A combined LME analysis detected significant main effects of Age-Group (*P* = 0.003) and MouseType (*P* = 0.022), as well as a significant interaction (*P* = 0.003), demonstrating that phase velocity trajectories differed between WT and *mdx* mice. Model estimates and WT–*mdx* contrasts are provided in Table S4–6 in the Supplementary Material. Across all ages, *mdx* mice exhibited higher phase velocities than WT, with gaps that widened at later stages. These findings complement the group-specific models and emphasize that both developmental and pathological factors contribute to the increases observed in *mdx* mice. Residual normality was assessed using the Anderson–Darling test for each phase velocity model. While most models showed no significant deviation from normality (*P* > 0.05), minor violations were observed for the G1 model (*P* = 0.050) and the combined WT model (*P* = 0.034). Given the small sample sizes and the mild nature of these deviations, the models were retained for analysis, as linear mixed-effects models are generally robust to slight departures from normality.

**Fig. 5.**
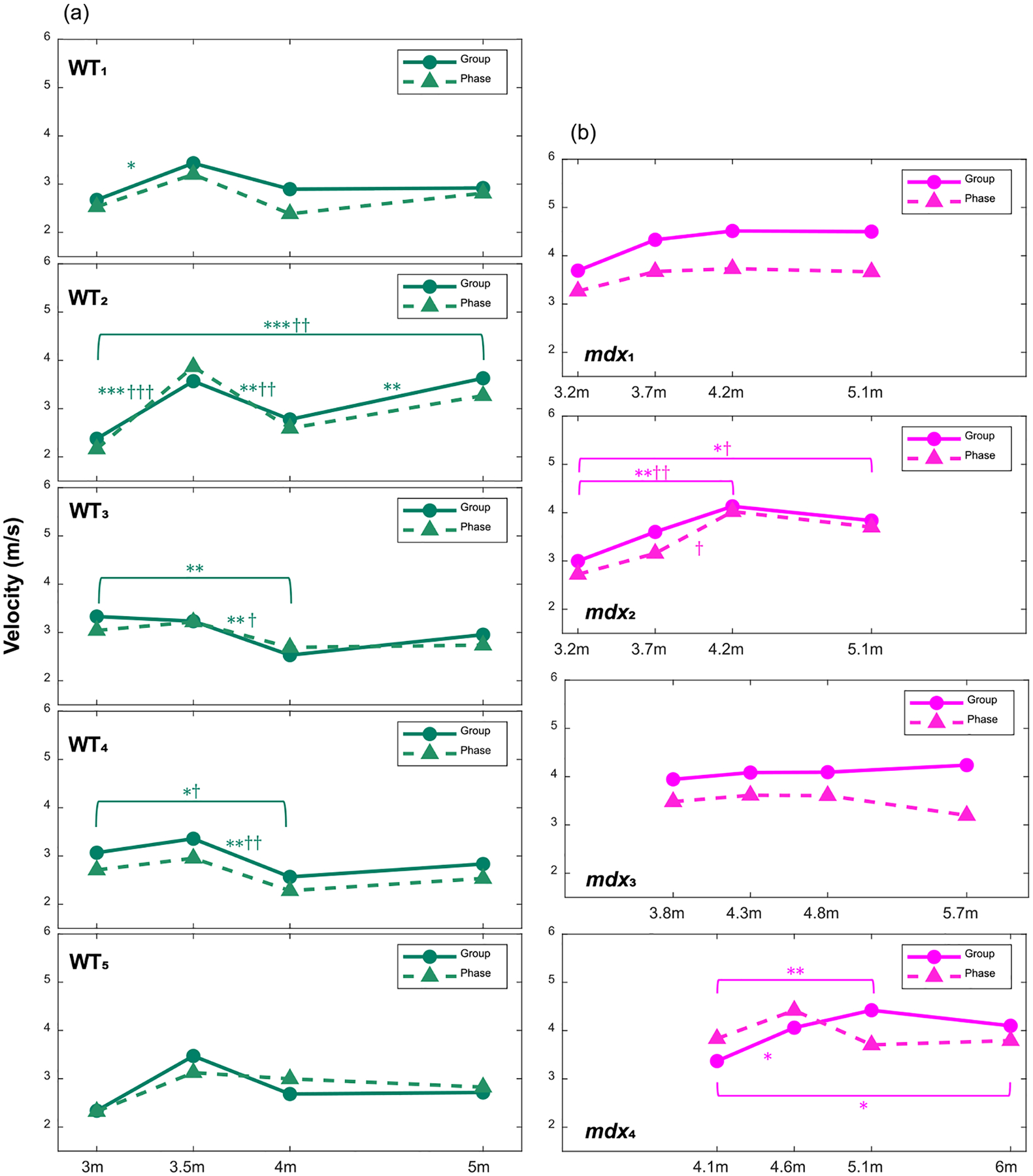
(a) Individual group (solid) and phase (dashed) velocity (m/s) measurements for WT mice aged 3 to 5 months (m). Significant age-related differences were observed in WT_2_, WT_3_, and WT_4_, particularly between 3 and 4 months. WT_1_ and WT_5_ exhibited relatively stable values across age groups, suggesting minimal age-related change. (b) Individual group velocity (m/s) measurements for *mdx* mice aged 3.2 to 6 months. *mdx*_2_ and *mdx*_4_ showed significant increases over time, whereas *mdx*_1_ and *mdx*_3_ maintained consistently elevated values across age. *P < 0.05, **P < 0.01, ***P < 0.001, ****P < 0.0001 (asterisks indicate significance in group velocity, dagger symbols (†) indicate significance in phase velocity).

Individual group velocity measurements for each mouse are displayed in Fig. 5, offering a detailed view of variability across animals that may be obscured in group-level averages. Among WT mice, significant changes over time were primarily observed in WT_2_, where increases were generally evident up to the final time point. WT_3_ and WT_4_ also showed noticeable variations between 3 and 4 months, suggesting individual differences in developmental trajectories of muscle mechanical properties. These findings indicate that while some WT mice exhibit relatively stable values across age groups, others experience age-related fluctuations in group velocity. In *mdx* mice, although early time points were unavailable for *mdx*_4_ (scanning began at 4.1 months), both *mdx*_2_ and *mdx*_4_ showed significant increases in velocity over time, aligning with the broader trend of progressive changes observed in the *mdx* model. In contrast, *mdx*_1_ and *mdx*_3_ showed no significant differences but maintained consistently elevated velocities. These findings suggest that increases in velocity during disease progression may occur either in the earlier stages (3–4 months) or later stages (after 4 months), likely reflecting a combination of normal development and disease-related changes.

**Fig. 6.**
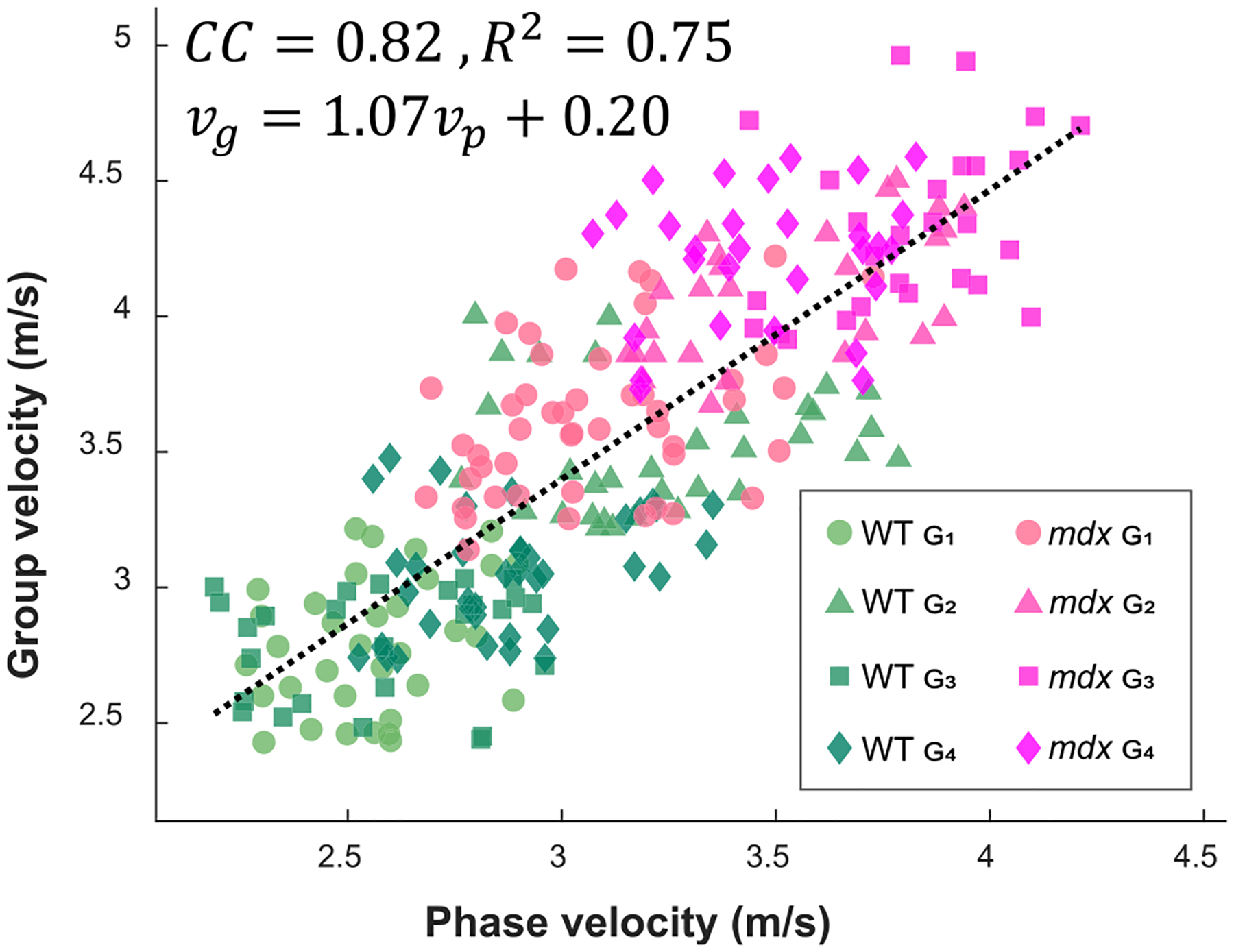
Correlation analysis and linear regression between phase velocity and group velocities. The phase velocity (*v*_*p*_) and corresponding group velocity (*v*_*g*_) showed a strong positive correlation (CC = 0.82; *R*^2^ = 0.75; *P* < 0.0001). The linear fit equation resulted in *v*_*g*_ = 1.07 *v*_*p*_ + 0.20.

Fig. 6 presents the correlation analysis and linear regression between phase velocity (*v*_*p*_) and group velocity (*v*_*g*_). This analysis was based on individual velocity measurements derived from two separate shear wave signals, rather than the median values used in earlier statistical comparisons. To minimize the impact of extreme measurements and emphasize the most representative data, values outside the 30th to 70th percentile range were excluded. The phase velocity values, extracted from the 600–900 Hz range, demonstrated a strong positive correlation with the corresponding group velocity (correlation coefficient, CC = 0.82; *R*^2^ = 0.75; *P* < 0.0001). The resulting linear fit equation was *v*_*g*_ = 1.07 *v*_*p*_ + 0.20, indicating a significant linear relationship between the two variables. These findings suggest that changes in the viscoelastic properties of the tissue are consistently reflected in both measurements, supporting the use of *v*_*g*_ and *v*_*p*_ to track similar underlying biomechanical changes.

### 3.3. Histology and correlation analysis

**Fig. 7.**
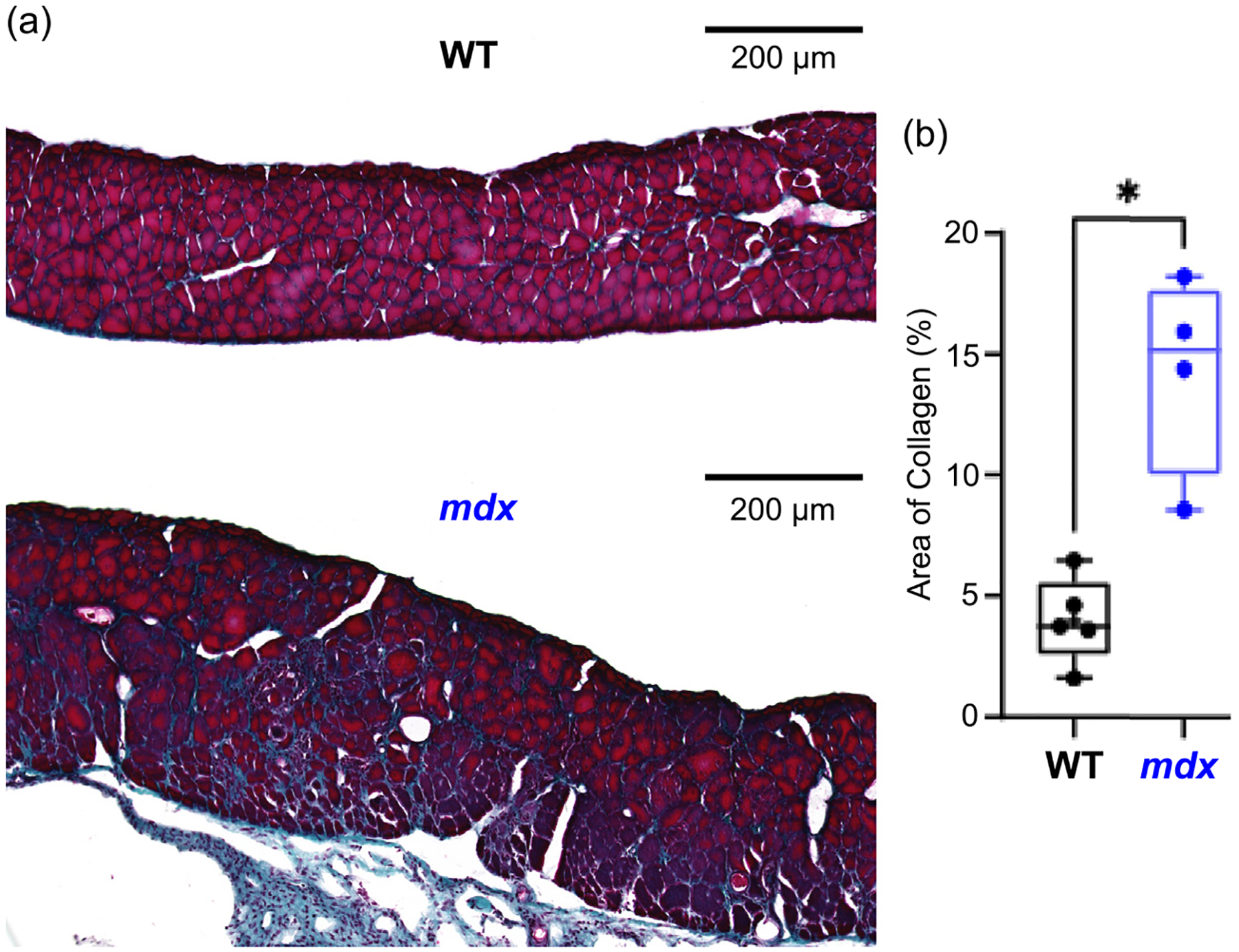
(a) Collagen deposition in WT and *mdx* mouse. In the trichrome stain, muscle fibers appear red/purple, while collagen is indicated in blue. Scale bar: 200 μm. (b) Quantification of collagen deposition area based on histological analysis. Box plots display 25th, 50th, and 75th percentiles, with whiskers extending to minimum and maximum values. * *P* < 0.05.

After ultrasound imaging at AgeGroup G4, the WT and *mdx* mice were euthanized, and collagen area was measured to assess fibrosis. Gomori’s Trichrome staining was used to quantify collagen deposition in the diaphragm. Dystrophic diaphragms from *mdx* mice exhibited greater collagen deposition compared to healthy WT diaphragms (Fig. 7). Quantification revealed that the percentage of collagen area was significantly lower in WT mice (4.0 ± 1.8%) than in *mdx* mice (14.3 ± 4.1%).

**Fig. 8.**
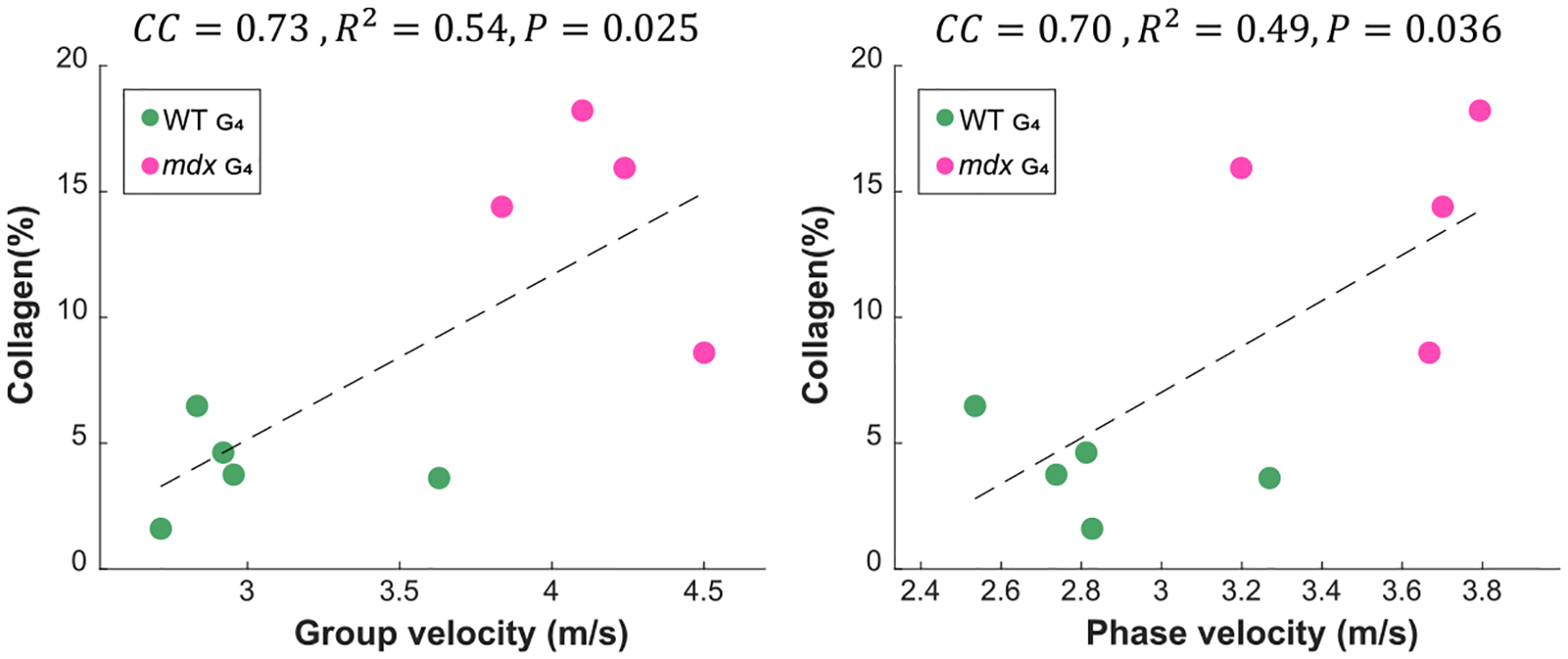
Correlation analysis and linear regression showing the relationship between collagen percentage (%) and (a) group velocity and (b) phase velocity in AgeGroup G4. Both analyses reveal a significant positive correlation (Group velocity: CC = 0.73, *R*^2^ = 0.54; *P* = 0.025; Phase velocity: CC = 0.70, *R*^2^ = 0.49; *P* = 0.036). The dashed line represents the linear regression fit.

The correlation analysis between *in vivo* shear wave measurements and quantified collagen from histology is shown in Fig. 8. For *in vivo* US-SWEI data, the mean of three measurements from each mouse was used as the representative value. The analysis revealed a statistically significant positive correlation between collagen deposition in the di-aphragm and both group and phase velocities (group velocity: CC = 0.73, *R*^2^ = 0.54, *P* = 0.025; phase velocity: CC = 0.70, *R*^2^ = 0.49, *P* = 0.036). These findings indicate that increased collagen deposition corresponds to higher shear wave group and phase velocities.

## 4. Discussion

The findings from this study highlight the feasibility and sensitivity of US-SWEI in detecting early-stage disease progression in *mdx* mice aged 3–6 months by measuring the viscoelastic properties of the diaphragm *in vivo*.

### 4.1. Early-stage disease progression in mdx mice

Functional deficits in the *mdx* diaphragm have been reported prior to overt structural damage, with reduced force output and altered passive mechanics contributing to impaired respiratory performance [44, 45]. This progression has been extensively characterized, including fibrosis development, collagen remodeling, and mechanical deterioration of the diaphragm muscle [46–48]. Experimental advances have also improved functional assessments in preclinical studies, enabling more precise characterization of diaphragm impairment in the *mdx* model [49,50]. These early mechanical abnormalities may underlie the stiffness alterations detected by US-SWEI and are consistent with histological findings in *mdx* mice.

Both group and phase velocity measurements demonstrated statistically significant changes throughout the scanning period in *mdx* mice, confirming their value in monitoring disease progression. Early-stage alterations (3–4 months) were especially evident, with some mice showing significant increases while others maintained consistently elevated values. These findings underscore the sensitivity of shear wave elastography to biomechanical changes in the diaphragm caused by Duchenne muscular dystrophy (DMD). These changes should be interpreted in the context of both natural developmental growth and disease progression, as both likely contribute to the observed trends. In WT mice, the transient pattern, with an increase from G1 to G2 followed by a decrease in G3 before rising again at G4, may reflect nonlinear aspects of post-maturation remodeling. Although structural maturation occurs earlier in development, skeletal muscle continues to undergo subtle adjustments in fiber size, extracellular matrix organization, hydration, and passive stiffness that can follow non-monotonic trajectories during postnatal remodeling [51–53]. By contrast, *mdx* mice displayed a more consistent increase across time points, consistent with progressive pathological remodeling, most notably fibrosis, dominating the mechanical phenotype.

It is important to note that histology was performed only at the terminal time point, which constrains interpretation of correlation analyses. The associations between shear wave metrics and collagen deposition therefore primarily reflect group-level differences at this late stage, rather than individual trajectories across time. While this limits the ability to track longitudinal progression directly, these findings remain informative as a reference point and may serve as a useful benchmark for evaluating treatment efficacy in preclinical studies.

### 4.2. Group vs. phase velocity

The strong correlation between group and phase velocities observed in this study demonstrates that both metrics effectively reflect changes in the viscoelastic properties of the diaphragm tissue. However, caution is needed when selecting the frequency range for wavelet-based phase velocity estimation to ensure accuracy, given the method’s inherent sensitivity to frequency band selection. In this work, phase velocity was analyzed within a standardized 600–900 Hz band to provide consistent comparisons across groups. This restriction improved stability of ridge extraction but reduced sensitivity to potentially informative lower-frequency behavior. Thus, the choice represents a trade-off between reliability and spectral coverage.

Additionally, due to the diaphragm’s thin, layered structure, the shear waves observed in this study may exhibit guided wave characteristics [54], a behavior known to influence both group and phase velocity estimations in soft tissues. Future studies should further characterize the wave mode to better interpret the frequency-dependent behavior resulting from guided wave propagation.

The slight differences in sensitivity between group and phase velocity suggest that they may capture distinct aspects of tissue mechanics. Phase velocity, influenced by tissue elasticity, viscosity, and structural characteristics, has the potential to provide complementary insights. In applications involving short propagation distances, such as the mouse diaphragm, group velocity offers a straightforward and accessible measure of tissue stiffness. The comparable correlations observed between group and phase velocities with collagen percentage suggest that group velocity alone may suffice for practical purposes, simplifying the assessment process without sacrificing accuracy. Nonetheless, phase velocity remains a valuable metric, particularly for capturing nuanced biomechanical properties that may complement group velocity measurements in specific contexts.

### 4.3 Contextualizing findings across age and disease

**Fig. 9.**
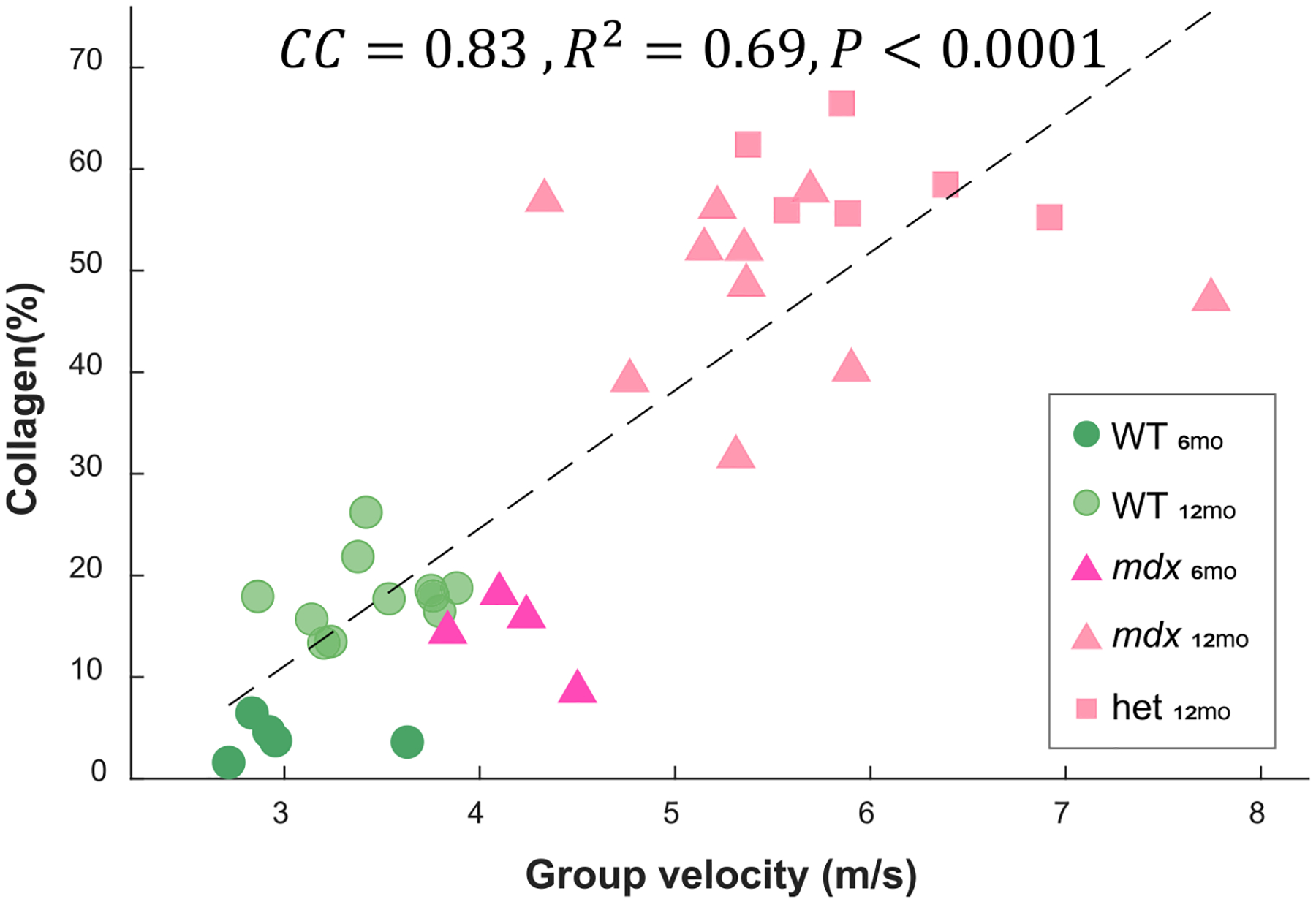
Correlation analysis between group velocity (m/s) and collagen deposition percentage (%) in young (6 mo.) and old (12 mo.) mice groups. Old mice include WT_12mo_, *mdx*_12mo_, and het_12mo_ (*mdx*/UTR-heterozygous), which is known for its severe pathology [55]. The dashed line represents the linear regression fit, showing a strong positive correlation across all data (CC = 0.83, *R*^2^ = 0.69, *P* < 0.0001).

Fig. 9 presents the correlation between group velocity and collagen deposition, combining results from this study and our previous study [24], where shear wave measurements and collagen percentages were analyzed in 12-month-old mice. In that earlier study, the group velocity was obtained between two points within a shorter segment (1–2 mm). For consistency with the current methodology, its data were reprocessed to match the current approach, where the median value is calculated from multiple individual measurements within a longer segment (2.5–3 mm). In older mice, excessive fibrosis leading to much higher group velocities contributed to the strong correlation (CC = 0.83; *R*^2^ = 0.69; *P* < 0.0001). However, in younger mice (WT_6mo_, *mdx*_6mo_) and in healthy but older mice (WT_12mo_), the sensitivity of group velocity to collagen percentage appears to be lower. This reduced sensitivity may stem from the limitations of shear waves, including variability and degraded stability caused by the structural features of the mouse diaphragm, which may overshadow the pathological changes in younger mice. Additionally, whereas collagen percentage in WT_12mo_ was relatively equivalent to *mdx*_6mo_, other factors inherent to aging – such as alterations in extracellular matrix composition, changes in muscle fiber elasticity, increased fat deposition, and reduced muscle tone – may influence shear wave velocity, contributing to the observed variability and the lower velocities compared to younger, dystrophic (*mdx*) mice.

Whereas this study provides promising findings, it has several limitations that warrant consideration and highlight areas for future research. The relatively small sample size may have affected robustness, with increased sensitivity to outliers and inter-animal variability in *mdx* mice. Repeatability was also not formally assessed; while all acquisitions followed a standardized protocol, intra- and inter-session reproducibility remain to be established and will be critical for translational work. SWE acquisitions were restricted to the costal diaphragm near the mid-sternal axis, which provides a relatively stable acoustic window for consistent measurements [56,57], but this approach may not fully capture heterogeneity between costal and crural regions. Within the costal region, fibers radiate outward from the central tendon toward the rib cage, so transverse slices may intersect fibers at oblique angles. Because shear wave velocity is influenced by anisotropy, this introduces potential variability, which we could not directly quantify since local fiber orientation could not be measured *in vivo*.

In addition, because the axial analysis window encompassed the full diaphragm thickness, our SWE estimates represent through-thickness averages rather than resolving sub-layer differences. Lateral averaging across analysis segments may also reduce sensitivity to patchy fibrosis. As a result, the measurements reflect global diaphragm mechanics, and higher-resolution SWE or histological co-registration would be required to resolve spatial heterogeneity. Another factor is the potential contribution of intramuscular fat, which can increase with disease progression and may exert effects opposite to those of fibrosis. Prior studies indicate that fat infiltration in the *mdx* diaphragm is generally minimal [58,59], consistent with our histological observations where collagen deposition was the dominant feature. Nevertheless, we did not perform quantitative fat analysis, and future studies with lipid-specific staining will be valuable to better disentangle the relative contributions of fibrosis and fat infiltration. Finally, physiological parameters such as airway pressure or EMG were not recorded, so subtle differences in diaphragm loading could have influenced velocities despite timing acquisitions to a late-exhalation window.

## 5. Conclusion

This study demonstrated the utility of ultrasound shear wave elasticity imaging (US-SWEI) for assessing diaphragm viscoelastic properties to monitor early-stage disease progression in *mdx* mice. Shear wave measurements sensitively captured biomechanical changes associated with Duchenne muscular dystrophy (DMD), offering a noninvasive means to detect disease-related alterations in diaphragm tissue. By targeting early-stage changes, this approach enables the identification of biomechanical markers that may inform therapeutic evaluation and development. These findings underscore the potential of US-SWEI as a valuable tool in preclinical research, providing quantitative and reproducible metrics for disease monitoring. Its noninvasive nature and responsiveness to tissue mechanical changes also suggest translational potential for clinical applications, supporting accessible and objective monitoring strategies for DMD and other neuromuscular disorders.

## Supplementary Material

1

## Figures and Tables

**Table 1:** WT group velocities (LME model and pairwise comparison).

Fixed effects coefficients				
AgeGroup	m/s			P
	Estimate	SE	95% CIs	
G1 (baseline)	2.76	0.11	[2.53 2.99]	**<0.0001**
G2	3.41	0.16	[2.86 3.97]	**0.0002**
G3	2.69	0.16	[2.14 3.25]	0.684
G4	3.01	0.16	[2.46 3.57]	0.124

Pairwise comparison	P
G1 vs. G2	**<0.0001**
G2 vs. G3	**<0.0001**
G3 vs. G4	0.05
G1 vs. G3	**<0.0001**
G1 vs. G4	**<0.0001**

**Table 2:** *Mdx* group velocities (LME model and pairwise comparison).

Fixed effects coefficients				
AgeGroup	m/s			P
	Estimate	SE	95% CIs	
G1 (baseline)	3.47	0.15	[3.17 3.77]	**<0.0001**
G2	3.96	0.24	[3.47 4.45]	0.051
G3	4.03	0.21	[3.61 4.46]	**0.011**
G4	4.20	0.20	[3.79 4.60]	**0.0007**

Pairwise comparison	P
G1 vs. G2	**<0.0001**
G2 vs. G3	0.766
G3 vs. G4	0.416
G1 vs. G3	**<0.0001**
G1 vs. G4	**<0.0001**

**Table 3:** Comparison of WT and *mdx* group velocities by AgeGroup.

Fixed effects coefficients					
AgeGroup	m/s				P
	WT (baseline)	*mdx*	SE	95% CIs for difference	
G1	2.76	3.47	0.23	[0.25 1.18]	**0.004**
G2	3.41	3.96	0.20	[0.13 0.96]	**0.012**
G3	2.69	4.03	0.19	[0.95 1.73]	**<0.0001**
G4	3.01	4.20	0.17	[0.84 1.53]	**<0.0001**

**Table 4:** WT phase velocities (LME model and pairwise comparison).

Fixed effects coefficients				
AgeGroup	m/s			P
	Estimate	SE	95% CIs	
G1 (baseline)	2.55	0.13	[2.28 2.82]	**<0.0001**
G2	3.27	0.19	[2.62 3.92]	**0.00004**
G3	2.59	0.19	[1.94 3.24]	0.851
G4	2.84	0.19	[2.19 3.49]	0.142

Pairwise comparison	P
G1 vs. G2	**<0.0001**
G2 vs. G3	**0.0006**
G3 vs. G4	0.199
G1 vs. G3	**<0.0001**
G1 vs. G4	**<0.0001**

**Table 5:** *Mdx* phase velocities (LME model and pairwise comparison).

Fixed effects coefficients				
AgeGroup	m/s			P
	Estimate	SE	95% CIs	
G1 (baseline)	3.05	0.17	[2.71 3.38]	**<0.0001**
G2	3.44	0.27	[2.89 3.98]	0.159
G3	3.93	0.24	[3.46 4.40]	**0.0005**
G4	3.61	0.23	[3.16 4.07]	**0.016**

Pairwise comparison	P
G1 vs. G2	**<0.0001**
G2 vs. G3	0.079
G3 vs. G4	0.171
G1 vs. G3	**<0.0001**
G1 vs. G4	**<0.0001**

**Table 6:** Comparison of WT and *mdx* phase velocities by AgeGroup.

Fixed effects coefficients					
AgeGroup	m/s				P
	WT (baseline)	*mdx*	SE	95% CIs for difference	
G1	2.55	3.05	0.18	[0.13 0.86]	**0.009**
G2	3.27	3.43	0.29	[−0.43 0.76]	0.576
G3	2.59	3.87	0.23	[0.81 1.76]	**<0.0001**
G4	2.84	3.61	0.18	[0.41 1.14]	**0.0003**

## Data Availability

Data will be made available on request.

## Appendix A. Supplementary data

Supplementary material related to this article can be found online at https://doi.org/10.1016/j.ultras.2025.107846.
