# Supplementary material for: Noninvasive longitudinal assessment of early-stage Duchenne muscular dystrophy: In vivo diaphragm imaging in mdx mice
Source: Ultrasonics. Author manuscript; Available in PMC 2026 Jun 19. (PMC13280731; doi:10.1016/j.ultras.2025.107846)
Supplement: 1 [file NIHMS2185361-supplement-1.pdf]

Supplementary Material

Noninvasive longitudinal assessment of early-stage Duchenne muscular dystrophy: *in vivo* diaphragm imaging in *mdx* mice

Jeehyun Lee, Nia O. Myrie, Woojin M. Han, Young C. Jang, Andrés J. García, Stanislav Emelianov

I. Analyzing dispersive waves in viscoelastic media using wavelet transform

Ultrasound shear wave elastography (SWE) is a non-invasive imaging technique used to quantify muscle stiffness by measuring the propagation velocity of shear waves within tissue. This velocity is directly related to tissue elasticity and the mechanical load applied to muscles [1]. In conventional implementations, SWE often relates shear wave velocity to shear modulus by assuming that tissue behaves as an infinite, homogeneous, isotropic, and purely elastic medium. These assumptions (e.g., $\mu = \rho \cdot v^2$, or $\mu = 3\rho \cdot v^2$ under incompressibility, where ρ is the assumed density) provide computational convenience and are widely adopted in commercial platforms and prior muscle studies [20–25], but they do not accurately represent skeletal muscle, which is anisotropic, viscoelastic, and finite in size. Because biological tissues exhibit frequency-dependent wave propagation, phase velocity varies with frequency, introducing potential errors in conventional SWE methods. To address this, dispersion analysis methods have been developed to estimate frequency-dependent phase velocity from shear wave data.

For phase velocity estimation, the two-dimensional Fourier transform (2D-FT) is commonly used to analyze spatiotemporal wave propagation in k-space [2]. However, 2D-FT and similar methods [3-6] require a large number of lateral spatial sampling points, making them less suitable for scenarios where shear waves propagate over short distances (Figure S2(a)). To address this limitation, wavelet-based techniques, particularly the continuous wavelet transform (CWT), have been employed to extract dispersion curves from signals recorded at closely spaced points. Originally developed in geophysics for interstation phase velocity analysis [7,8], CWT has been adapted for shear wave phase velocity estimation in biological tissues [9]. This approach effectively compensates for limited spatial sampling, allowing mechanical property assessment even when shear wave propagation distances are small. However, the CWT suffers from resolution trade-offs due to the wavelet's shape, leading to smearing, leakage, and reduced precision at lower frequencies [10-12]. The Synchrosqueezing Transform (SST) refines the CWT by redistributing spectral energy in the time-frequency domain, improving resolution and making it better suited for analyzing complex, non-stationary signals [13-15].

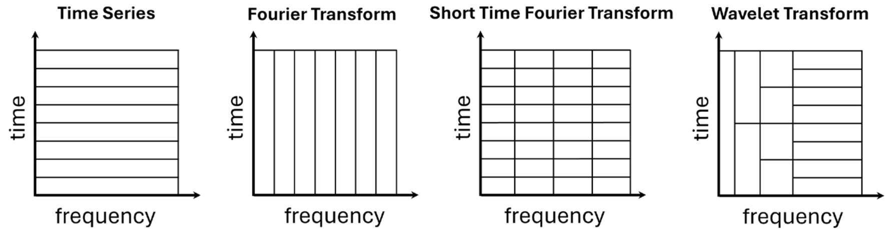

Fig. S1. Comparison of time and frequency decomposition methods: FT retains only frequency information; STFT provides limited time localization with fixed windows; Wavelet Transform captures both frequency and precise time localization with variable windows.

<u>*Continuous Wavelet Transform*</u>

The Fourier Transform (FT) decomposes signals into their frequency components but assumes stationarity, making it unsuitable for analyzing time-varying signals. The Short-Time Fourier Transform (STFT) attempts to address this by applying FT over fixed time windows, enabling localized frequency analysis. However, the fixed window size in STFT imposes a trade-off between time and frequency resolution. The Continuous Wavelet Transform (CWT) mitigates this issue by employing scalable wavelets, which dynamically adapt to different frequency components, providing high time resolution for fast-varying signals and high frequency resolution for slowly changing components (Figure S1). Mathematically, the CWT is defined as:

$$W(\tau,\alpha) = \frac{1}{\sqrt{\alpha}}\int_{-\infty}^{+\infty} s(t)\varphi^*\left(\frac{t-\tau}{\alpha}\right)dt \tag{1}$$

where, τ represents the time shift, α the scale, and $*$ denotes a complex conjugation. In this work, the analytic Morlet wavelet is used as the mother wavelet, defined as:

$$\varphi(\omega) = 2e^{-\frac{(\omega-6)^2}{2}}H(\omega) \tag{2}$$

where $H(\omega)$ is the unit step function in the frequency domain.

Synchrosqueezing Wavelet Transform

The Synchrosqueezing Transform (SST) is an extension of the CWT that enhances the time-frequency representation (TFR) by concentrating energy around instantaneous frequencies. This method improves the sharpness of the time-frequency plot, making frequency variations more distinguishable, especially in signals with dynamic frequency content. Unlike standard CWT, which suffers from energy smearing, SST refines the representation by reassigning wavelet coefficients to their true frequency locations, leading to a more focused TFR. By extracting instantaneous frequency information and redistributing the wavelet coefficients accordingly, the SST provides a highly concentrated spectral representation [14-18]. This makes it particularly useful for analyzing complex, real-world signals [10], including those found in ultrasound elastography and biomedical applications. The SST assumes that the input signal $s(t)$ consists of multiple oscillatory components with time-dependent amplitudes and phases, modeled as:

$$s(t) = \sum_{k=1}^{K} A_k(t)\cos\big(\theta_k(t)\big), \tag{3}$$

where K is the number of signal components, A_k and θ_k are the time-varying amplitude and phase of the kth signal component [11]. The goal of the SST is to retrieve the instantaneous frequencies $f_k = \frac{1}{2\pi}\frac{d\theta_k(t)}{dt}$ for each component and reassign the energy in the CWT at each time τ and scale α to these frequencies. To achieve this, we start from the CWT of $s(t)$, defined in Equation (3). By differentiating the phase of the CWT with respect to τ, we obtain the instantaneous frequency $f(\tau,\alpha)$:

$$f(\tau,\alpha) = -\frac{1}{2\pi}\frac{\mathrm{Im}(\frac{\partial W(\tau,\alpha)}{\partial \tau})}{W(\tau,\alpha)}, \tag{4}$$

where $\mathrm{Im}(\cdot)$ denotes the imaginary part. This frequency estimation allows the redistribution of wavelet coefficients according to precise frequency values, enhancing clarity in the time-frequency domain. The synchrosqueezed transform $T(\tau,\alpha)$ then transfers energy from the scale-time domain (τ,α) to the time-frequency domain (τ,f), mapping each CWT coefficient to its calculated frequency:

$$T(\tau,\alpha) = \int W(\tau,\alpha)\delta\big(f - f(\tau,\alpha)\big)\frac{d\alpha}{\alpha}. \tag{5}$$

Here, $\delta(f - f(\tau,\alpha))$ is the Dirac delta function, ensuring that only energy corresponding to frequency $f(\tau,\alpha)$ is retained at each point. This concentration procedures a sharper, more readable time-frequency representation, beneficial for analyzing complex biological signals in ultrasound elastography.

Phase velocity estimation using wavelet transform

To evaluate phase velocity dispersion, synthetic shear wave signals were generated to simulate wave propagation in soft tissue. The initial wave signal w_1 at $x = 0\ mm$ was modeled as a Gaussian-modulated sinusoidal wave:

$$w_1(t) = Ae^{-(t-t_0)^2}\sin(2\pi f_0 t)$$

where the central frequency $f_0 = 500\,Hz$ and a time offset $t_0 = 2ms$ (Fig S2.(a)). To simulate shear wave propagation, an additional signal w_2 was generated by introducing dispersion effects over a predefined propagation distance (Figure S2(b)). The empirical dispersion model used in the simulation follows the A0 mode of Lamb waves [19]:

$$v_p(f) = \sqrt{\frac{2\pi f D {c_s}^2}{2\sqrt{3}}},$$

where D is the thickness of the medium (set to $D = 2mm$) and c_s is the shear wave speed. Signal w_2 represents w_1 propagated to $x = 5\,mm$ with a phase velocity v_p where $c_s = 3\,m/s$. The wavelet analysis was conducted using MATLAB (Wavelet Toolbox, MathWorks, Natick, MA, USA). The magnitude $|W|$ and phase arg (W) spectra for w_1 and w_2 are shown in Figures S2(b, e) and (c, f), respectively. To improve visualization, the SST magnitude spectrum was filtered with a median filter to reduce peak artifacts. For phase velocity estimation, the frequency band was selected based on a 6 dB attenuation threshold, covering a range of 193–846 Hz. Figure S3(a) presents the stacked cross-correlograms of the phase spectra for w_1 and w_2, with the dashed black line indicating the time shift $t_{ph}(f)$ for each frequency. The final phase velocity curve (Figure S3(b)) was computed as:

$$v_{p,estimated}(f) = \frac{x}{t_{ph}(f)}$$

This method ensures accurate dispersion analysis for shear wave propagation in biological tissues.

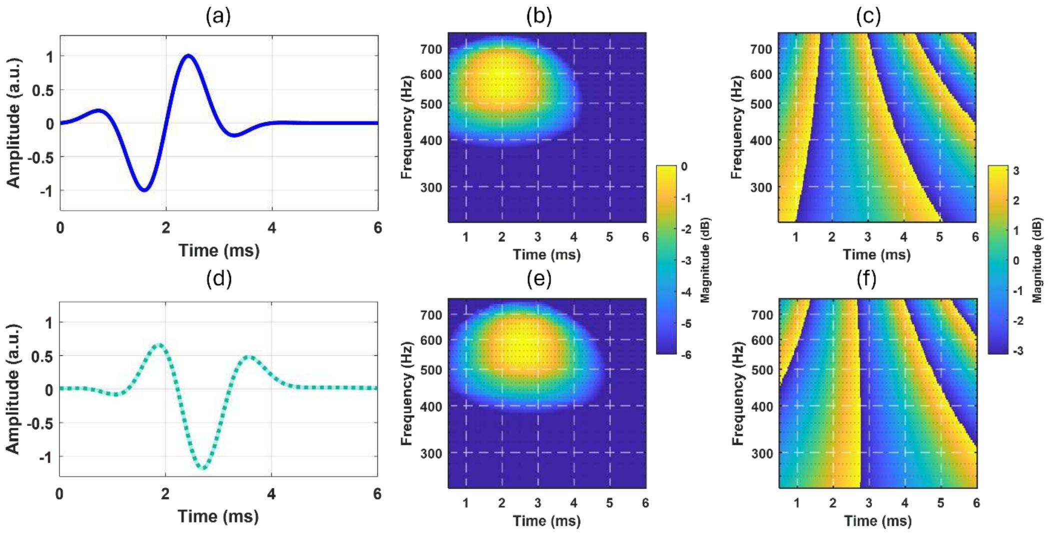

Fig. S2. Wave propagation and frequency analysis. (a) The initial wave signal w_1 at $x = 0\ mm$. **(b)** Magnitude spectrum for w_1. **(c)** Phase spectrum for w_1. **(d)** Propagated wave w_2 at $x = 5\ mm$. **(e)** Magnitude spectrum for w_2 **(f)** Phase spectrum for w_2

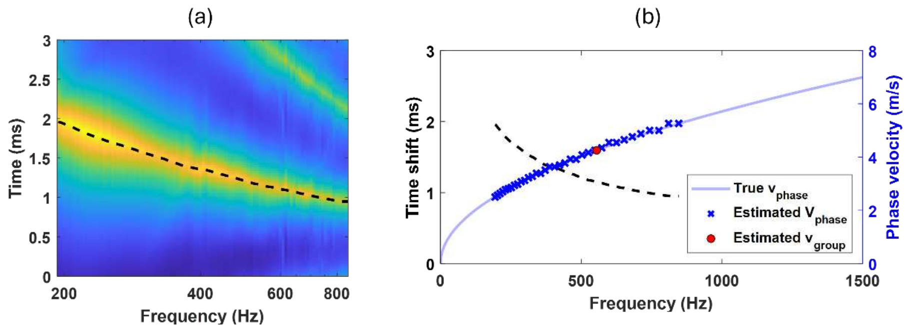

Fig. S3. (a) Stacked cross-correlograms of the phase spectra of w_1 and w_2. The dashed black line represents the maximal time lags used to estimate the time-shift. **(b)** Phase velocity estimation.

II. Combined linear mixed-effects model analysis

a. Group velocity

Table S1. Fixed effects from the combined LME model

Effect	Estimate	SE	95% CI	P value
Intercept (WT, G1)	2.76	0.13	[2.49, 3.02]	**<0.0001**
MouseAge G2	0.66	0.19	[0.28, 1.03]	**0.0007**
MouseAge G3	-0.07	0.19	[-0.44, 0.31]	0.724
MouseAge G4	0.25	0.19	[-0.12, 0.63]	0.181
MouseType (*mdx*)	0.71	0.19	[0.34, 1.09]	**0.0002**
MouseAge G2 × MouseType (*mdx*)	-0.17	0.29	[-0.74, 0.40]	0.558
MouseAge G3 × MouseType (*mdx*)	0.63	0.27	[0.10, 1.15]	**0.020**
MouseAge G4 × MouseType (*mdx*)	0.47	0.26	[-0.04, 0.99]	0.072

Table S2. Estimated marginal means (EMMs) from the combined LME model

AgeGroup	WT (m/s)	95% CI (WT)	*mdx* (m/s)	95% CI (*mdx*)
G1	2.76	[2.49, 3.02]	3.47	[3.21, 3.74]
G2	3.41	[3.15, 3.68]	3.96	[3.62, 4.30]
G3	2.69	[2.43, 2.95]	4.03	[3.77, 4.30]
G4	3.01	[2.75, 3.27]	4.20	[3.96, 4.44]

Table S3. WT–*mdx* contrasts from the combined LME model

AgeGroup	Difference (m/s)	SE	95% CI	P value
G1	0.71	0.19	[0.35, 1.08]	**0.0002**
G2	0.55	0.22	[0.12, 0.97]	**0.013**
G3	1.34	0.19	[0.97, 1.71]	**<0.0001**
G4	1.19	0.18	[0.84, 1.54]	**<0.0001**

b. Phase velocity

Table S4. Fixed effects from the combined LME model

Effect	Estimate	SE	95% CI	P value
Intercept (WT, G1)	2.55	0.15	[2.25, 2.85]	<0.0001
MouseAge G2	0.72	0.21	[0.30, 1.14]	0.0010
MouseAge G3	0.04	0.21	[-0.39, 0.46]	0.867
MouseAge G4	0.28	0.21	[-0.14, 0.71]	0.188
MouseType (*mdx*)	0.49	0.21	[0.07, 0.92]	0.022
MouseAge G2 × MouseType (*mdx*)	-0.33	0.33	[-0.98, 0.31]	0.311
MouseAge G3 × MouseType (*mdx*)	0.84	0.30	[0.24, 1.44]	0.006
MouseAge G4 × MouseType (*mdx*)	0.28	0.30	[-0.30, 0.87]	0.343

Table S5. Estimated marginal means (EMMs) from the combined LME model

AgeGroup	WT (m/s)	95% CI (WT)	***mdx*** **(m/s)**	**95% CI (*****mdx*****)**
G1	2.55	[2.25, 2.85]	3.05	[2.75, 3.35]
G2	3.27	[2.97, 3.57]	3.44	[3.05, 3.82]
G3	2.59	[2.29, 2.89]	3.92	[3.63, 4.22]
G4	2.84	[2.54, 3.14]	3.61	[3.34, 3.89]

Table S6. WT–*mdx* contrasts from the combined LME model

AgeGroup	Difference (m/s)	SE	95% CI	P value
G1	0.50	0.21	[0.08, 0.91]	0.022
G2	0.16	0.25	[-0.32, 0.65]	0.508
G3	1.34	0.21	[0.92, 1.75]	<0.0001
G4	0.78	0.20	[0.38, 1.18]	<0.0001

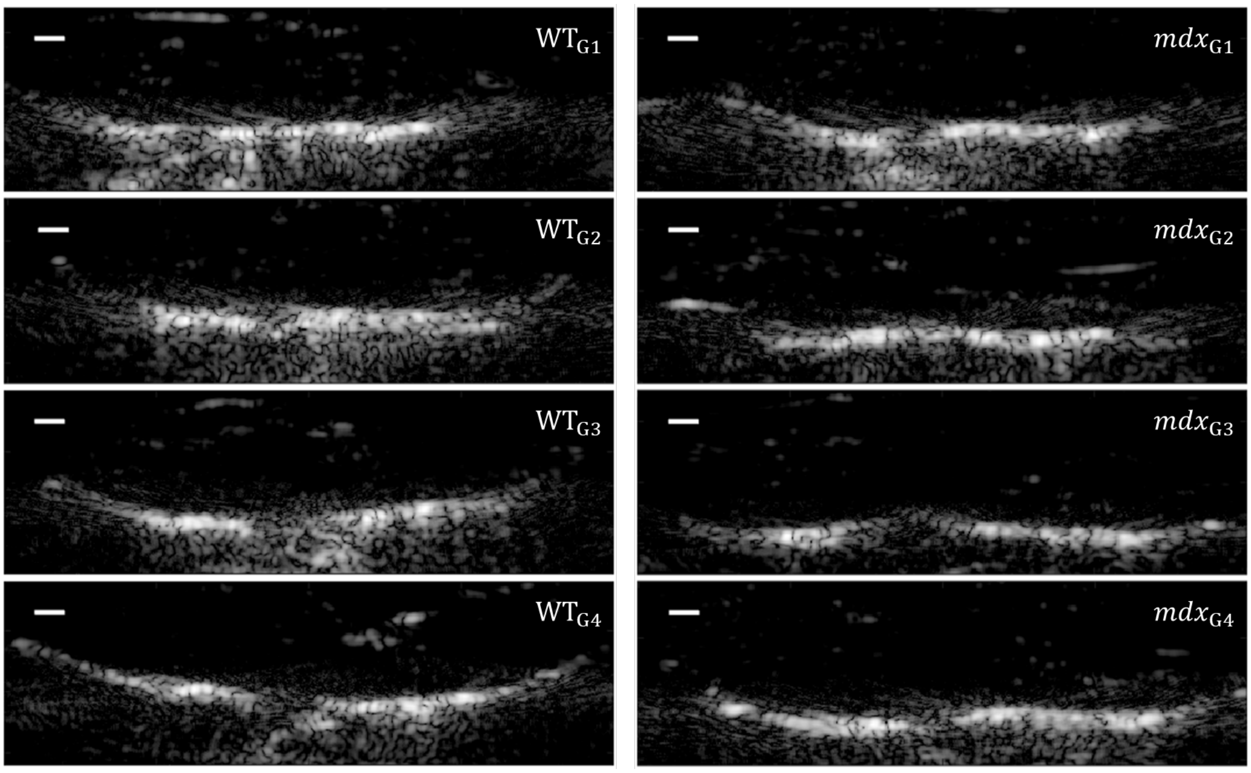

Fig. S4. Representative B-mode ultrasound images of the diaphragm. Left column: WT mice; right column: *mdx* mice. Each column shows one representative image from age groups G1–G4 (top to bottom).

REFERENCES

[1] A. Nordez and F. Hug, "Muscle shear elastic modulus measured using supersonic shear imaging is highly related to muscle activity level," J. Appl. Physiol., vol. 108, no. 5, pp. 1389–1394, 2010. [Online]. Available: https://doi.org/10.1152/japplphysiol.01323.2009.

[2] D. N. Alleyne and P. Cawley, "A two-dimensional Fourier transform method for the measurement of propagating multimode signals," J. Acoust. Soc. Am., vol. 89, pp. 1159–1168, 1991.

[3] S. McAleavey, M. Menon, and E. Elegbe, "Shear modulus imaging with spatially-modulated ultrasound radiation force," Ultrason. Imaging, vol. 31, no. 4, pp. 217–234, 2009. [Online]. Available: https://doi.org/10.1177/016173460903100401.

[4] K. R. Nightingale et al., "Derivation and analysis of viscoelastic properties in human liver: Impact of frequency on fibrosis and steatosis staging," IEEE Trans. Ultrason. Ferroelectr. Freq. Control, vol. 62, no. 1, pp. 165–175, Jan. 2015. [Online]. Available: https://doi.org/10.1109/TUFFC.2014.006653.

[5] L. Ambrozinski et al., "Identification of material properties – efficient modelling approach based on guided wave propagation and spatial multiple signal classification," Struct. Control Health Monit., vol. 22, no. 7, pp. 969–983, 2015. [Online]. Available: https://doi.org/10.1002/stc.1728.

[6] P. Kijanka et al., "Robust phase velocity dispersion estimation of viscoelastic materials used for medical applications based on the multiple signal classification method," IEEE Trans. Ultrason. Ferroelectr. Freq. Control, vol. 65, no. 3, pp. 423–439, Mar. 2018. [Online]. Available: https://doi.org/10.1109/TUFFC.2018.2792324.

[7] Q. Wu et al., "Measurement of interstation phase velocity by wavelet transformation," Earthq. Sci., vol. 22, no. 4, pp. 425–429, Aug. 2009. [Online]. Available: https://doi.org/10.1007/s11589-009-0425-3.

[8] S. Mao et al., "On the measurement of seismic traveltime changes in the time–frequency domain with wavelet cross-spectrum analysis," Geophys. J. Int., vol. 221, no. 1, pp. 550–568, 2019. [Online]. Available: https://doi.org/10.1093/gji/ggz495.

[9] P. Kijanka, L. Ambrozinski, and M. W. Urban, "Two-point method for robust shear wave phase velocity dispersion estimation of viscoelastic materials," Ultrasound Med. Biol., vol. 45, no. 9, pp. 2540–2553, Sep. 2019. [Online]. Available: https://doi.org/10.1016/j.ultrasmedbio.2019.04.016.

[10] M. Sylvain, O. Thomas, and P. Duong-Hung, "Synchrosqueezing transforms: From low- to high-frequency modulations and perspectives," Comptes Rendus Phys., vol. 20, no. 5, pp. 449–460, 2019. [Online]. Available: https://doi.org/10.1016/j.crhy.2019.07.001.

[11] J. B. Tary, R. H. Herrera, and M. van der Baan, "Analysis of time-varying signals using continuous wavelet and synchrosqueezed transforms," Philos. Trans. A Math. Phys. Eng. Sci., vol. 376, no. 2126, Aug. 2018. [Online]. Available: https://doi.org/10.1098/rsta.2017.0254.

[12] D. Zhang, Fundamentals of Image Data Mining: Analysis, Features, Classification and Retrieval. Springer, 2019.

[13] S. Maes and I. Daubechies, "A nonlinear squeezing of the continuous wavelet transform based on auditory nerve models," in Proc. Int. Conf. Acoust. Speech Signal Process. (ICASSP), 2017, p. 527.

[14] I. Daubechies, J. Lu, and H.-T. Wu, "Synchrosqueezed wavelet transforms: An empirical mode decomposition-like tool," Appl. Comput. Harmon. Anal., vol. 30, no. 2, pp. 243–261, 2011. [Online]. Available: https://doi.org/10.1016/j.acha.2010.08.002.

[15] Z. Liu et al., "Synchrosqueezed wavelet transform-based method for characterizing the dispersive nature of laser-excited surface acoustic waves propagating through the coated or damaged medium," Measurement, vol. 185, p. 109965, 2021. [Online]. Available: https://doi.org/10.1016/j.measurement.2021.109965.

[16] H.-T. Wu, P. Flandrin, and I. Daubechies, "One or two frequencies? The synchrosqueezing answers," Adv. Data Sci. Adapt. Anal., vol. 3, pp. 29–39, 2011.

[17] R. H. Herrera, J. Han, and M. van der Baan, "Applications of the synchrosqueezing transform in seismic time-frequency analysis," Geophysics, vol. 79, no. 3, pp. V55–V64, 2014. [Online]. Available: https://doi.org/10.1190/geo2013-0204.1.

[18] B. Ryan et al., "The synchrosqueezing transform to evaluate paleoclimate cyclicity," Comput. Geosci., vol. 175, p. 105336, 2023. [Online]. Available: https://doi.org/10.1016/j.cageo.2023.105336.

[19] T.-M. Nguyen et al., "Assessment of viscous and elastic properties of sub-wavelength layered soft tissues using shear wave spectroscopy: Theoretical framework and in vitro experimental validation," IEEE Trans. Ultrason. Ferroelectr. Freq. Control, vol. 58, pp. 2305–2315, Nov. 2011. [Online]. Available: https://doi.org/10.1109/TUFFC.2011.2088.

[20] Bercoff, Jérémy et al. "Supersonic shear imaging: a new technique for soft tissue elasticity mapping." IEEE transactions on ultrasonics, ferroelectrics, and frequency control vol. 51,4 (2004): 396-409. doi:10.1109/tuffc.2004.1295425

[21] Creze, Maud et al. "Shear wave sonoelastography of skeletal muscle: basic principles, biomechanical concepts, clinical applications, and future perspectives." Skeletal radiology vol. 47,4 (2018): 457-471. doi:10.1007/s00256-017-2843-y

[22] Goo, Miran et al. "Systematic Review of Instrumented Measures of Skeletal Muscle Mechanical Properties: Evidence for the Application of Shear Wave Elastography with Children." Ultrasound in medicine & biology vol. 46,8 (2020): 1831-1840. doi:10.1016/j.ultrasmedbio.2020.04.009

[23] Olchowy, Cyprian et al. "Stiffness of the Masseter Muscle in Children-Establishing the Reference Values in the Pediatric Population Using Shear-Wave Elastography." International journal of environmental research and public health vol. 18,18 9619. 13 Sep. 2021, doi:10.3390/ijerph18189619

[24] Pichiecchio, Anna et al. "Muscle ultrasound elastography and MRI in preschool children with Duchenne muscular dystrophy." Neuromuscular disorders : NMD vol. 28,6 (2018): 476-483. doi:10.1016/j.nmd.2018.02.007

[25] Lin, Chia-Wei et al. "Acoustic radiation force impulse shear wave elastography quantifies upper limb muscle in patients with Duchenne muscular dystrophy." Ultrasonics sonochemistry vol. 101 (2023): 106661. doi:10.1016/j.ultsonch.2023.106661
